# Comparison of the effects of moderate and severe hypercapnic acidosis on ventilation-induced lung injury

**DOI:** 10.1186/s12871-015-0050-8

**Published:** 2015-04-30

**Authors:** Wanchao Yang, Ziyong Yue, Xiaoguang Cui, Yueping Guo, Lili Zhang, Huacheng Zhou, Wenzhi Li

**Affiliations:** 1 Department of Anesthesiology, Second Affiliated Hospital of Harbin Medical University; Anesthesiology Key Laboratory, Harbin Medical University, Harbin, 150086 China; 2 Education Department of Heilongjiang Province, Anesthesiology Key Laboratory, Harbin Medical University, Harbin, Heilongjiang Province China

**Keywords:** Hypercapnic acidosis, Ventilation-induced lung injury, Nuclear factor kappa B, Cytokines

## Abstract

**Background:**

We have proved that hypercapnic acidosis (a PaCO_2_ of 80-100 mmHg) protects against ventilator-induced lung injury in rats. However, there remains uncertainty regarding the appropriate target PaCO_2_ or if greater CO_2_ “doses” (PaCO_2_ > 100 mmHg) demonstrate this effect. We wished to determine whether severe acute hypercapnic acidosis can reduce stretch-induced injury, as well as the role of nuclear factor-κB (NF-κB) in the effects of acute hypercapnic acidosis.

**Methods:**

Fifty-four rats were ventilated for 4 hours with a pressure-controlled ventilation mode set at a peak inspiratory pressure (PIP) of 30 cmH_2_O. A gas mixture of carbon dioxide with oxygen (FiCO_2_ = 4-5%, FiCO_2_ = 11-12% or FiCO_2_ = 16-17%; FiO_2_ = 0.7; balance N_2_) was immediately administered to maintain the target PaCO_2_ in the NC (a PaCO_2_ of 35-45 mmHg), MHA (a PaCO_2_ of 80-100 mmHg) and SHA (a PaCO_2_ of 130-150 mmHg) groups. Nine normal or non-ventilated rats served as controls. The hemodynamics, gas exchange and inflammatory parameters were measured. The role of NF-κB pathway in hypercapnic acidosis-mediated protection from high-pressure stretch injury was then determined.

**Results:**

In the NC group, high-pressure ventilation resulted in a decrease in PaO_2_/FiO_2_ from 415.6 (37.1) mmHg to 179.1 (23.5) mmHg (p < 0.001), but improved by MHA (379.9 ± 34.5 mmHg) and SHA (298.6 ± 35.3 mmHg). The lung injury score in the SHA group (7.8 ± 1.6) was lower than the NC group (11.8 ± 2.3, P < 0.05) but was higher than the MHA group (4.4 ± 1.3, P < 0.05). Compared with the NC group, after 4 h of high pressure ventilation, the MHA and SHA groups had decreases in MPO activity of 67% and 33%, respectively, and also declined the levels of TNF-α (58% versus 72%) and MIP-2 (76% versus 60%) in the BALF. Additionally, both hypercapnic acidosis groups reduced stretch–induced NF-κB activation (p < 0.05) and significantly decreased lung ICAM-1 expression (p < 0.05).

**Conclusions:**

Moderate hypercapnic acidosis (PaCO_2_ maintained at 80-100 mmHg) has a greater protective effect on high-pressure ventilation-induced inflammatory injury. The potential mechanisms may involve alterations in NF-κB activity.

## Background

Mechanical ventilation with high pressure or high volume has been reported that can induce lung injury as the typical of hyaline membrane formation, pulmonary edema, and deterioration in oxygenation [1,2]. Dreyfuss et al. reported that ventilation with 45 cm H_2_O peak inspiratory pressure (PIP) resulted in pathophysiological changes in the lungs, such as the destruction of epithelial lining and basement membrane [1]. Recent researches have focused on trying to decrease mortality by reducing ventilator-induced lung injury (VILI) [3,4]. These attempts impose restrictions on the tidal volume (V_T_) and inflation pressure and may lead to hypercapnic acidosis (HA). Studies have also found that hypercapnic acidosis can directly attenuate experimental acute lung injuries induced by ischemia-reperfusion [5], free radicals [6], endotoxin [7,8], systemic sepsis [9,10], and VILI both *ex vivo* [11,12] and *in vivo* [12-14]. These studies indicated that hypercapnic acidosis may reduce lung injury, with the mechanisms hypothesized to function through anti-inflammatory and lung surfactant effects [15] as well as a reduction in NO [16] and attenuation of the nuclear factor kappa B (NF-κB) pathway [12]. NF-κB is a key transcriptional factor that modulates the gene expression of various pro-inflammatory cytokines and adhesion molecules [17-19]. More recent studies have demonstrated that the effects of HA—both beneficial and deleterious—may be mediated at least in part via the inhibition of NF-κB activity [12,20].

In our previous study, the protective effects of hypercapnic acidosis associated with a particular level of PaCO_2_ have been demonstrated in moderate-range hypercapnic acidosis (PaCO_2_ maintained at 80-100 mmHg) [21]. Although the dose-response characteristics of hypercapnic acidosis have previously been demonstrated in the setting of ischemia-reperfusion injury [22], there remains uncertainty regarding the appropriate target PaCO_2_ in the setting of VILI *in vivo*. Our previous results from an in vivo model of cerebral ischemia showed that a PaCO_2_ of 100 mmHg may be the upper limit of the neuroprotective range of hypercapnia [23]. This evidence indicates that higher doses of PaCO_2_ likely had adverse effects on neurologic outcomes in a rat cerebral ischemia model. Whether this phenomenon is similar to that in high-pressure ventilation-induced lung injury is less clear.

We hypothesized that greater CO_2_ “doses” (PaCO_2_ > 100 mmHg) in rats receiving high-pressure ventilation (HPV) were associated with decreased protective effects of HA in reducing pulmonary inflammatory injury. We sought: (1) to compare the effects of moderate hypercapnic acidosis (PaCO_2_ of 80-100 mmHg) and severe hypercapnic acidosis (PaCO_2_ of 130-150 mmHg) on HPV-induced inflammatory injury and (2) to elucidate the role of the NF-κB pathway in this process.

## Methods

### Experimental protocol

The experimental protocols were approved by the Institutional Animal Care and Use Committee of Harbin Medical University, and conducted in compliance with the animal-use guidelines (SYXK (Hei) 2006-033). Seventy-two adult Wistar rats (weight 250-300 g) were anesthetized with an intraperitoneal injection of 30 mg/kg of pentobarbital sodium. The internal carotid artery was cannulated with a 20-gauge catheter to aspirate blood for blood gas analysis and arterial pressure monitoring. The rectal temperature was maintained at 37.0-38.5°C. Mechanical ventilation delivered via tracheostomy was initiated in the pressure-controlled mode (Kent Scientific Ventilator-Dual Mode, USA) with 15 cmH_2_O PIP, a positive end-expiratory pressure (PEEP) of 2 cmH_2_O, a frequency of 30 breaths/min to maintain the PaCO_2_ at 35-45 mmHg, an inspiration-to-expiration (I:E) ratio of 1:2, and an inhaled oxygen fraction (FiO_2_) of 0.7 for 15 min, after which baseline data were collected.

Prior to randomization, the following values needed to be stable: PaO_2_/FiO_2_ > 300 mmHg, PaCO_2_ 30-45 mmHg, and HCO_3_^-^ > 20 mmol · L^-1^. If any parameter was not fulfilled, the animals were excluded from the protocol and further data analysis.

### Experimental groups

Seventy two rats were randomly assigned to 8 blocks of 9 animals each, with random numbers generated by SPSS (version 13.01S; Beijing Stats Data Mining Co. Ltd, Beijing, China). Among them, two blocks were randomly assigned to the sham group (anaesthetized and non-ventilated rats) and NV group (ventilated with PIP = 15 cmH_2_O and inhaled FiO_2_ of 0.7 for 4 h) served as controls for assessing the expression of NF-κB p65 protein and the inflammatory mediators in the lung. The left 6 blocks were assigned to three groups through merging two blocks of rats randomly, and including the Normocapnia (NC) group (PaCO_2_ = 35-45 mmHg, n = 18), PaCO_2_ was maintained in the normal range through inhaling the gas mixture (FiO_2_ 0.7, FiCO_2_ 4-5%, balance N_2_); the Moderate Hypercapnic Acidosis (MHA) group (PaCO_2_ = 80-100 mmHg, n = 18), PaCO_2_ was maintained through inhaling the gas mixture (FiO_2_ 0.7, FiCO_2_ 11-12%, balance N_2_); and the Severe Hypercapnic Acidosis (SHA) group (PaCO_2_ = 130-150 mmHg, n = 18), PaCO_2_ was maintained through inhaling the gas mixture (FiO_2_ 0.7, FiCO_2_ 16-17%, balance N_2_). The rats in the three groups were ventilated for 4 h in the supine position with a PIP of 30 cmH_2_O via the pressure-controlled mode (inspiratory time = 0.7 s; PEEP = 2 cmH_2_O and respiratory rate = 30 breaths/min). For all rats, **a**nesthesia was maintained with sodium pentobarbital (2-4 mg · kg^-1^ · hr^-1^) and pancuronium bromide (0.03-0.07 mg · kg^-1^ · hr^-1^). Throughout the experiment, frequent checks were made to ensure that the animals were adequately anesthetized. This was performed by applying a painful stimulus to a paw and observing blood pressure responses. Lactated Ringer’s solution was infused i.v. at 10 ml · kg^-1^ · hr^-1^ to compensate for blood sampling.

### Measurement of physiologic indices

In all experimental series, the systemic mean blood pressure (MAP) and heart rate (HR) were recorded at baseline, initiation of test conditions, and at 1-hour intervals thereafter. These were measured using an MP150 Workstation and analyzed using the AcqKnowledge software (BIOPAC Systems, Inc., Santa Barbara, CA) according to the manufacturer’s specifications. Tidal volumes (V_T_) were determined every 60 min with a VT Plus HF Gas Flow Analyzer (Fluke Corporation, USA). Inhaled and exhaled CO_2_ and O_2_ were tested using a gas monitor (DATEX Instrumentarium, Helsinki, Finland). Arterial blood samples were taken at baseline and every 60 min after randomization in each series, and blood gas analysis was performed (Rapidlab 248, Bayer Company, USA).

### Assay of inflammatory mediators in BALF and myeloperoxidase activity in lungs

At the end of the experiment, animals were exsanguinated, and the heart and lungs were dissected from the thorax. The right lobe bronchus was lavaged using sterile saline with 5 ml of saline (0.9%, 4°C) by three separate washes, and 4 ml of bronchoalveolar lavage fluid (BALF) was collected. A 1.0 ml aliquot was used for cell counts. The remaining fluid was centrifuged (300 × g at 4°C for 10 min), and the cell-free supernatant was divided into two 1-ml aliquots. One aliquot was snap-frozen in liquid nitrogen and stored at -80°C for subsequent analysis of tumor necrosis factor (TNF-a), interleukin (IL)-1β, and macrophage inflammatory protein-2 (MIP-2) using a commercial enzyme-linked immunosorbent assay kits (R&D Systems, Minneapolis, MN, USA). The remaining aliquot was frozen at -20°C for a measurement of the total protein concentration (BCA; Pierce, Rockford, IL). The right lobe of the lung was stored at -80°C and was later ground into homogenate to measure myeloperoxidase (MPO) activity using a kit (Jiancheng Bio-Technology, Nanjing, China) and a spectrophotometer. One unit of MPO was defined as the quantity that degraded 1.0 mmol of peroxide per minute at 37°C. The results were expressed as units per gram of wet lung tissue (U/g).

### Histology and immunohistochemistry

A 1 cm^3^ core sample was extracted from the visually estimated center of the left upper lobe of the lung, fixed in 4% buffered formalin and embedded in paraffin. The samples were then sectioned, stained with hematoxylin and eosin, and examined by a pathologist who was blinded to the protocol. The evaluation was based on the following criteria as described previously [24]: (1) neutrophil infiltration; (2) interstitial edema; (3) alveolar edema; (4) hyaline membrane formation. Each criterion was scored on a semiquantitative scale of 0-4, where 0 = normal, 1 = minimal change, 2 = mild change, 3 = moderate change and 4 = severe change. An overall histological score was calculated by totaling the scores for criteria 1 through 4. The left lower lobe of the lung was used to determine the wet: dry weight ratio (WW: DW) of the lung.

The expression of intercellular adhesion molecule-1 (ICAM-1) and NF-κB in lung sections was measured by immunohistochemical staining with an ICAM-1 detection kit (Zhongshan Golden Bridge Biotechnology, Beijing, China) and an NF-κB p65 antibody (Santa Cruz Biotechnology, Inc., Santa Cruz, CA). Specific labeling was detected with an Elite ABC peroxidase kit and diaminobenzidine (DAB) (Zhongshan Golden Bridge Biotechnology). Briefly, slides were systematically scanned at a lower magnification to define the lung injury by evaluating H&E-stained slides along with consecutive immunohistochemistry-stained slides. Eight to ten representative digital images were acquired from each slide using a 40× objective. Brown granules were quantified as positively stained cells or nuclei in each high-powered field (400 x magnification). The results were expressed as the percentage of positively stained cells or ratio of nuclei to total cells from 8–10 digital images per animal and four animals per group.

### Determination of NF-κB and IκB-α concentration

Tissues were homogenized in RIPA buffer and lysed for 30 min on ice. Samples were then sonicated, vortexed and centrifuged at 12,000 × g for 20 min at 4°C. Nuclear and cytoplasmic fractionation was performed using an EZ nuclei isolation kit (Applygen Technologies Inc.; China). Nuclear P65 concentrations and cytoplasmic IκB-α concentrations were determined using western blot analysis. Briefly, the supernatants were collected and separated using SDS-polyacrylamide gels, blotted onto membranes and incubated with the primary rabbit polyclonal anti-NF-κB p65 antibody, rabbit polyclonal anti-I-kappaB Kinase (IκB)-α antibody or ICAM-1 (M-19) antibody (Santa Cruz Biotechnology Inc., USA). Signals on the membranes were detected with an Odyssey Infrared Imaging System (LI-COR Bioscience, USA). The level of measured materials was normalized to the level of β-actin. Total lung NF-κB activity was determined using an activated NF-κB ELISA assay kit (TransAM NF-κB; Active Motif, Carlsbad, CA). In this kit, an oligonucleotide containing an NF-κB consensus site (5′-GGGACTTTCC-3′) was absorbed onto polystyrene microwells.

### Statistical analysis

All data are presented as mean (SD). Statistical analyses were performed using SPSS (version 13.01S; Beijing Stats Data Mining Co. Ltd, Beijing, China). Power calculations were performed prior to the commencement of the study. A sample size of 8 in each block will be sufficient to detect a difference of 0.1 U/g in MPO between the treatment and the control groups assuming a standard deviation of 0.1 U/g as reported in this population, at 80% power and 5% level of significance. This number has been increased to 9 per block (total of 72) to allow for a predicted drop-out from treatment of around 10%. Group comparisons were evaluated with a one-way ANOVA followed by the Student–Newman–Keuls (SNK) test for multiple comparisons. MAP, HR, V_T_, pH, PaCO_2_ and PaO_2_/FiO_2_ levels were evaluated by repeated measures ANOVA with time (five levels: baseline, at 1 hour, 2 hours, 3 hours and 4 hours after ventilation) as a within-subject factor and group (three levels: NC, MHA and SHA) as a between-subject factor. Overall significant differences in time, group and interaction between time and group were determined by a two-tailed p < 0.05.

## Results

Seven rats (two from each HPV group and one from NV group) that died before the 3 h mark were excluded from the study because of progressive hypotension. Forty-eight rats in HPV groups and eight rats in NV group survived the 4 h ventilation protocol and were included in the subsequent data analysis. The physiological characteristics of the NC, MHA and SHA groups were similar at baseline.

### Hemodynamic variables and arterial blood gas analysis

The hemodynamics induced by the VILI process and management are shown in Table 1. The V_T_ decreased gradually during the last 2 h period compared with the first 2 h period in the NC and SHA groups, but this effect was not observed in the MHA group (Table 1).

**Table 1:** **Hemodynamic parameters and gas exchange at different timepoints for each group**

	Baseline	1 h	2 h	3 h	4 h
MAP(mmHg)					
NC	126 ± 8	124 ± 10	119 ± 12	113 ± 12*	90 ± 24*
MHA	122 ± 13	130 ± 12	134 ± 9*^†^	135 ± 12*^†^	127 ± 12^†^
SHA	127 ± 15	137 ± 7*^†^	134 ± 7^†^	123 ± 12^†#^	113 ± 17*^†#^
HR(1/min)					
NC	347 ± 26	324 ± 31	295 ± 38*	283 ± 39*	258 ± 28*
MHA	362 ± 15	323 ± 17	294 ± 23*	283 ± 20*	270 ± 19*
SHA	347 ± 24	284 ± 29*^†#^	258 ± 40*^†#^	243 ± 38*^†#^	224 ± 35*^†#^
V_T_(ml)					
NC	2.4 ± 0.3	9.5 ± 0.9	9.2 ± 0.7	8.4 ± 1.0	5.9 ± 1.3*
MHA	2.4 ± 0.2	10.1 ± 0.9	9.9 ± 1.1	9.4 ± 0.9	8.8 ± 1.3^†^
SHA	2.4 ± 0.2	9.9 ± 0.7	9.7 ± 0.9	9.2 ± 0.9	7.8 ± 1.3*
PaO_2_/FiO_2_(mmHg)					
NC	361.4 ± 33.3	415.6 ± 37.1	426.9 ± 32.7	270.1 ± 48.8*	179.1 ± 23.5*
MHA	355.3 ± 39.5	428.6 ± 36.1*	448.4 ± 35.9*	432.2 ± 44.2*^†^	379.9 ± 34.5^†^
SHA	350.1 ± 34.8	456.8 ± 40.6*^†^	439.6 ± 25.2*	388.8 ± 49.8^†#^	298.6 ± 35.3*^†#^
pH					
NC	7. 42 ± 0.10	7. 39 ± 0.03	7. 37 ± 0.04	7. 32 ± 0.03*	7. 29 ± 0.08*
MHA	7. 39 ± 0.08	7. 10 ± 0.02*^†^	7. 07 ± 0.04*^†^	7. 07 ± 0.04*^†^	7. 06 ± 0.04*^†^
SHA	7. 37 ± 0.06	6. 94 ± 0.04*^†#^	6. 94 ± 0.03*^†#^	6. 93 ± 0.04*^†#^	6. 93 ± 0.03*^†#^
PaCO_2_ (mmHg)					
NC	41. 3 ± 10.1	36. 5 ± 4.0	40. 6 ± 4.8	43.0 ± 5.2	47.8 ± 9.6*
MHA	38. 7 ± 9.3	88. 7 ± 8.8*^†^	95. 1 ± 9.9*^†^	97.0 ± 9.5*^†^	93.6 ± 12.1*^†^
SHA	41. 7 ± 7.3	140.1 ± 12.6*^†#^	141.6 ± 11.1*^†#^	144.2 ± 14.7*^†#^	142.8 ± 16.7*^†#^

*Values are means ± SD; n = 16.*

*NC = high-pressure ventilation with a peak inspiratory pressure (PIP) of 30 cmH*_*2*_*O with normocapnia (inhaled 4*-*5% CO*_*2*_*to maintain Paco*_*2*_ *= 35*-*45 mmHg); MHA = NC plus moderate hypercapnic acidosis (inhaled 11*-*12% CO*_*2*_*to maintain Paco*_*2*_ *= 80*-*100 mmHg); SHA = NC plus severe hypercapnic acidosis (inhaled 16*-*17% CO*_*2*_*to maintain Paco*_*2*_ *= 130*-*150 mmHg); MAP = systemic mean blood pressures; HR = heart rates; PaO*_*2*_*/FiO*_*2*_ *= ratio of arterial oxygen tension to fraction of the inspired oxygen; PaCO*_*2*_ *= arterial carbon dioxide partial pressure; V*_*T*_ = *tidal volume.*

*p < 0.05 versus baseline except V_*T*_ *(versus 1 h);* ^*†*^ *p < 0.05 versus NC group;* ^*#*^ *p < 0.05 versus MHA group.*

The results of the blood gas analysis are shown in Table 1, including the PaO_2_/FiO_2_, PaCO_2_ and pH values. In the NC group, high-pressure ventilation resulted in a decrease in PaO_2_/FiO_2_ from 415.6 (37.1) mmHg to 179.1 (23.5) mmHg (p < 0.001). This gradually increased and was significantly improved in both hypercapnic acidosis groups (p < 0.05), but the values of PaO_2_/FiO_2_ in the MHA group were significantly higher than those in the SHA group at the end of the protocol (p < 0.05).

### Pulmonary permeability changes and neutrophil counts

**Figure 1:**
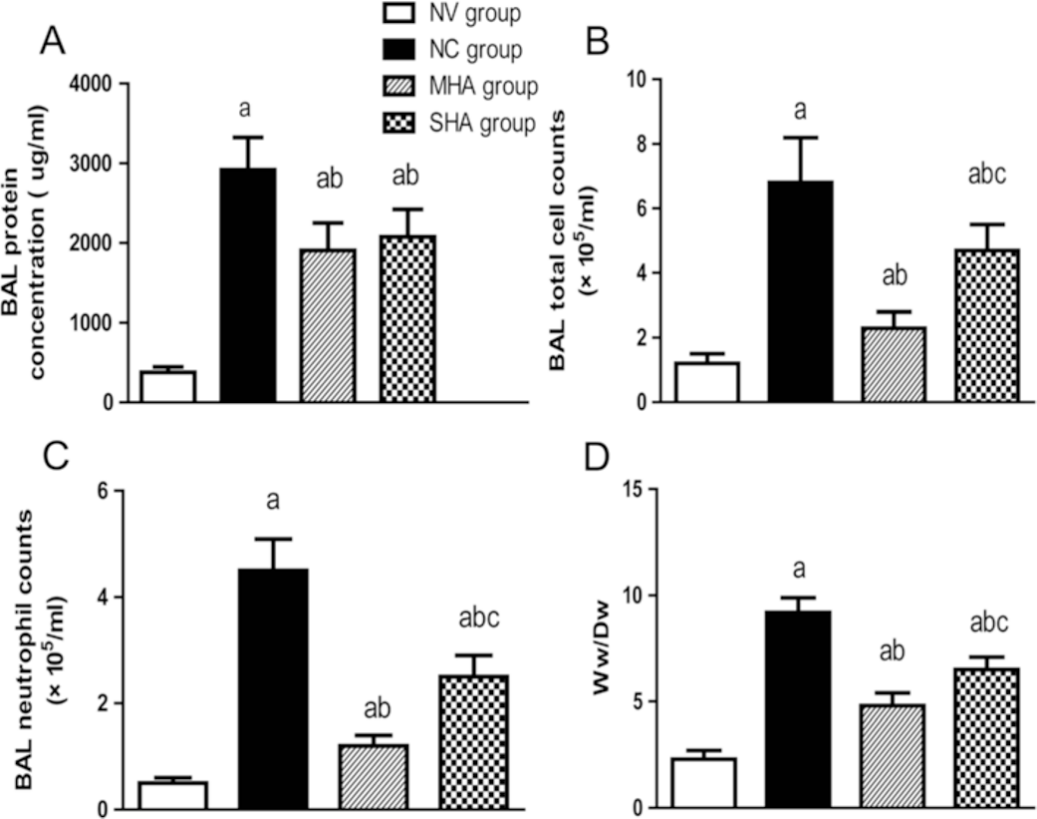
Effect of hypercapnic acidosis on major markers in BALF in rats’ lungs induced by high-pressure ventilation (HPV) for 4 h. **(A)** Bronchoalveolar lavage fluid (BALF) total protein levels (microgram/ml) were augmented by high-pressure ventilation but diminished by both hypercapnic groups. **(B, C)** The total and neutrophil cell count in the lavage group increased following HPV but were attenuated in the hypercapnic groups. **(D)** Pulmonary edema formation was quantified by measuring the wet and desiccated dry weights of the lung tissue. *n = 8 for each group.*^a^*p < 0.05 versus the NV group;*^b^*p < 0.05 versus the NC group;*^c^*p < 0.05 in the SHA group versus the MHA group.*

As shown in Figure 1, BALF protein concentrations and the WW: DW in both hypercapnic acidosis groups were significantly reduced compared with the NC group (p < 0.05). Inhalation of CO_2_ during VILI significantly decreased the total cell counts and neutrophil counts at the end of the experiment, and moderate hypercapnic acidosis had a greater effect than severe hypercapnic acidosis.

### Cytokines in BALF and myeloperoxidase activity in lung tissues

The levels of TNF-α and MIP-2 in the BALF were much lower in the NV group, but both significantly increased in the NC group (Table 2, p < 0.05). Hypercapnic acidosis attenuated TNF-α and MIP-2 levels in both HA groups (p < 0.05). After 4 h of high pressure ventilation, the MHA and SHA groups had decreases in MPO activity of 67% and 33%, respectively, compared with the NC group (Table 2).

**Table 2:** **Comparison of the level of TNF-a, IL-1β, and MIP-2 in BALF; MPO activity in lungs among groups**

	NV group	NC group	MHA group	SHA group
TNF-a (pg/ml)	64 ± 18	335 ± 107*	141 ± 49*^†^	93 ± 33*^#^
IL-1β (pg/ml)	223 ± 76	1571 ± 421*	709 ± 205*^†^	973 ± 224*^†^
MIP-2 (pg/ml)	74 ± 19	479 ± 114*	113 ± 31*^†^	190 ± 51*^†#^
MPO (U/g)	0.3 ± 0.1	1.2 ± 0.4*	0.4 ± 0.1^†^	0.8 ± 0.2*^†#^

*Values are means ± SD; n = 8.*

*NV = normal-pressure ventilation with a peak inspiratory pressure (PIP) of 15 cmH*_*2*_*O; NC = high-pressure ventilation with a peak inspiratory pressure (PIP) of 30 cmH*_*2*_*O; MHA = NC plus moderate hypercapnic acidosis (inhaled 11*-*12% CO*_*2*_*to maintain Paco*_*2*_ *= 80*-*100 mmHg); SHA = NC plus severe hypercapnic acidosis (inhaled 16*-*17% CO*_*2*_*to maintain Paco*_*2*_ *= 130*-150 mmHg); BALF, bronchoalveolar lavage fluid; *p < 0.05 versus NV group;^*†*^*p < 0.05 versus NC group;*^*#*^*p < 0.05 versus MHA group.*

### Histology

**Figure 2:**
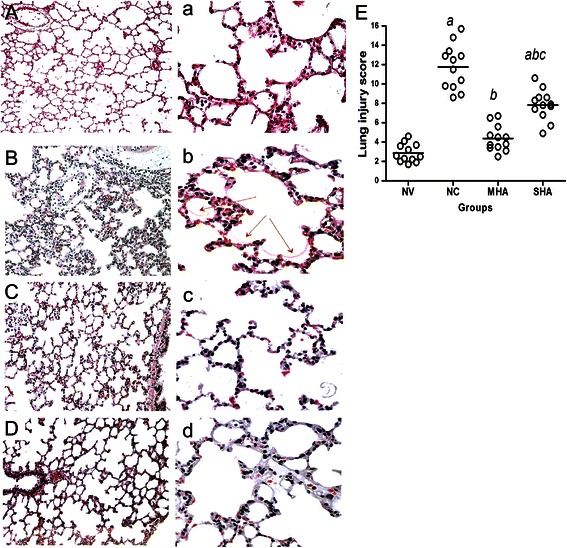
Histologic analysis of lungs. **(A and a)** NV group; **(B and b)** NC group; **(C and c)** MHA group; **(D and d)** SHA group and **(E)** lung injury scores in the four groups. NV = normal-pressure ventilation with normocapnia; NC = high-pressure ventilation with normocapnia; MHA = moderate hypercapnic acidosis; SHA = severe hypercapnic acidosis. The **A**, **B**, **C** and **D** panels represent 100x magnification, and the a, b, c and d panels represent 400x magnification. Severe edema in the alveolar septum and spaces with hyaline membrane formation (red arrow) were seen in the NC group, and rare neutrophil infiltration, moderate interstitial edema and less hyaline membrane formation were observed in both hypercapnic acidosis groups. Horizontal bars represent the median. *n = 12 for each group.*^*a*^*p* <0.05 versus the *NV* group; ^*b*^*p* <0.05 versus the *NC* group; ^*c*^*p < 0.001 in the SHA group versus the MHA group.*

Compared with the NV group (Figure 2A), microscopic findings in the lungs from NC rats (Figure 2B) showed moderate to severe edema in the alveolar septum and spaces, hyaline membrane formation. Much less severe changes were present in lungs from both hypercapnic acidosis groups (Figure 2C and D). The lung injury score in the NC group (11.8 ± 2.3) was higher than that in the NV group (2.9 ± 0.9, P < 0.001). SHA group (7.8 ± 1.6) was lower than the NC group (P < 0.05) but was higher than the MHA group (4.4 ± 1.3, P < 0.05) (Figure 2E).

### Lung ICAM-1 expression

**Figure 3:**
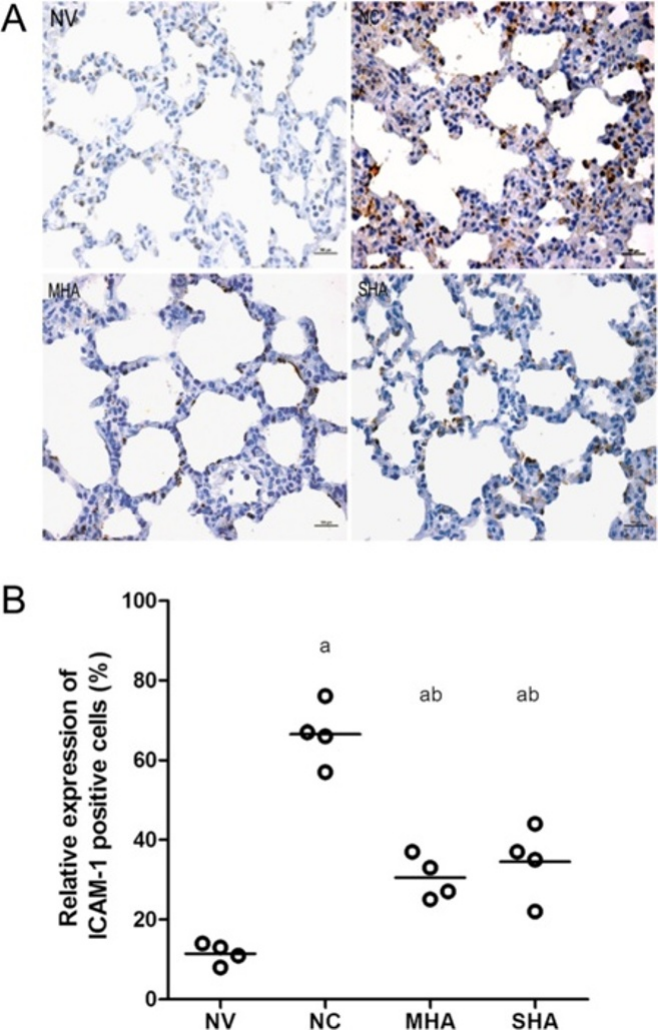
Lung immunohistochemistry for ICAM-1 protein expression in lung tissues. NV = normal-pressure ventilation with normocapnia; NC = high-pressure ventilation with normocapnia; MHA = moderate hypercapnic acidosis; SHA = severe hypercapnic acidosis **(A)** Immunohistochemical staining of ICAM-1 in the lung. Sections were stained with a brown DAB color-developing agent and counterstained with hematoxylin. The presence of brown granules in the nucleus was defined as a positive cell. All panels represent a 400x magnification. **(B)** Scatter plot of ICAM-1-positive (+) cells (%) in lung tissue. Semi-quantitative analysis of ICAM-1 indicated that hypercapnic acidosis decreased the expression of ICAM-1. Horizontal bars represent the median. *n = 4 for each group*. ^*a*^*p* <0.05 versus the *NV* group; ^*b*^*p* < 0.05 versus the *NC* group.

**Figure 4:**
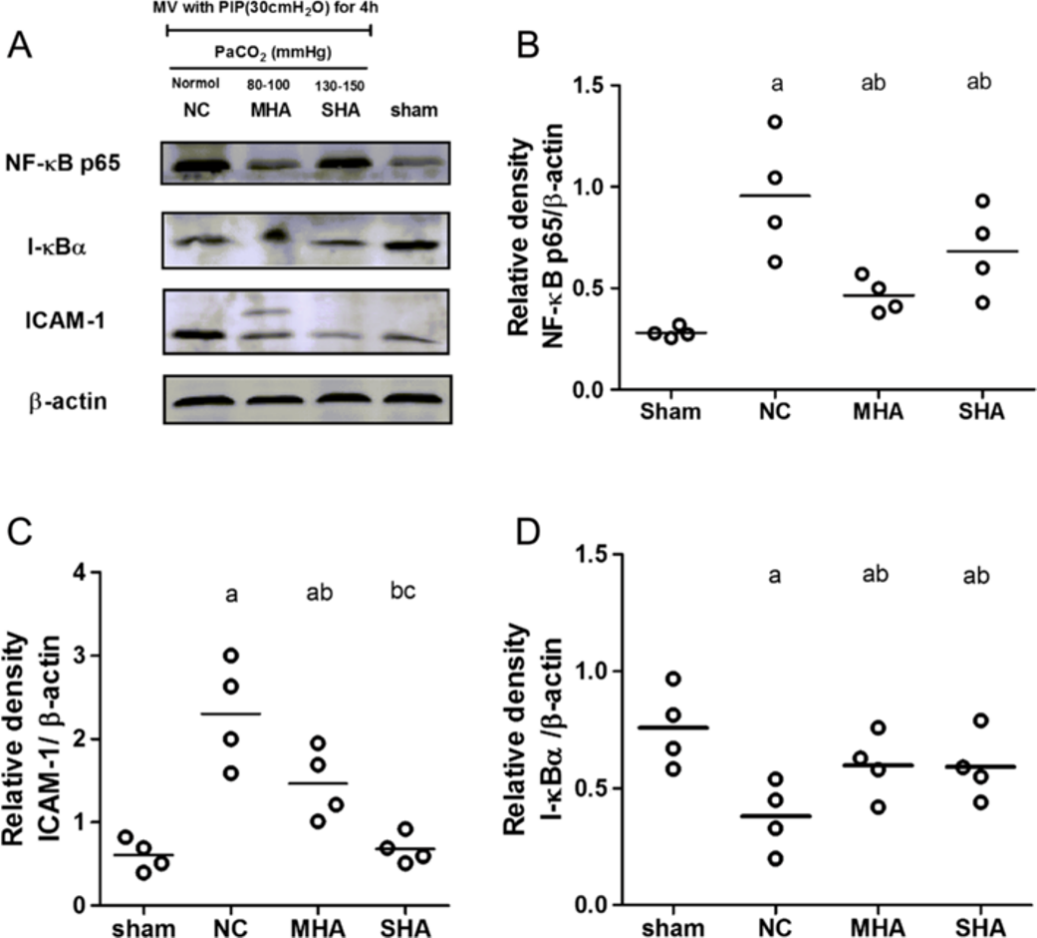
Western blot analysis of the nuclear p65, cytoplasmic IκB-α and ICAM-1 protein in lung tissues. sham = anaesthetized and non-ventilated rats; NC = high-pressure ventilation with normocapnia; MHA = moderate hypercapnic acidosis; SHA = severe hypercapnic acidosis. **(A)** A representative western blot of lung tissue nuclear p65, cytoplasmic IκBα and ICAM-1 from a sham (unventilated) animal, and animals exposed to HPV under HCA and normocapnic conditions. Both moderate and severe hypercapnic acidosis reduced nuclear p65 expression after 4 h of HPV (*p < 0.05*). IκB-α fractions were only reduced by HPV and were protected by the use of hypercapnic acidosis (*p < 0.05*). ICAM-1 showed positive expression, but this was highest in animals treated with only HPV. Densitometric readings of the western blot expressed as NF-κB and IκB-α and ICAM-1/loading control β-actin (panels **B**, **C** and **D**). Horizontal bars represent the median. *n = 4 for each group*. ^a^*p < 0.05 versus the sham group;*^b^*p < 0.05 versus the NC group;*^c^*p < 0.001 in the SHA group versus the MHA group.*

The presence of ICAM-1 as assessed by immunostaining significantly increased in the NC group compared with the normal ventilation group (Figure 3A and B). Hypercapnic acidosis apparently inhibited ICAM-1 expression (Figure 3A and B), but significant differences were not observed between the MHA and SHA groups (Figure 3A and B). Western blot analysis of ICAM-1 levels also revealed an increase in expression in the NC group compared to sham animals, and a reduction in ICAM-1 was observed in both HA groups (Figure 4A and C).

### Lung NF-κB expression and IκB-α degradation

**Figure 5:**
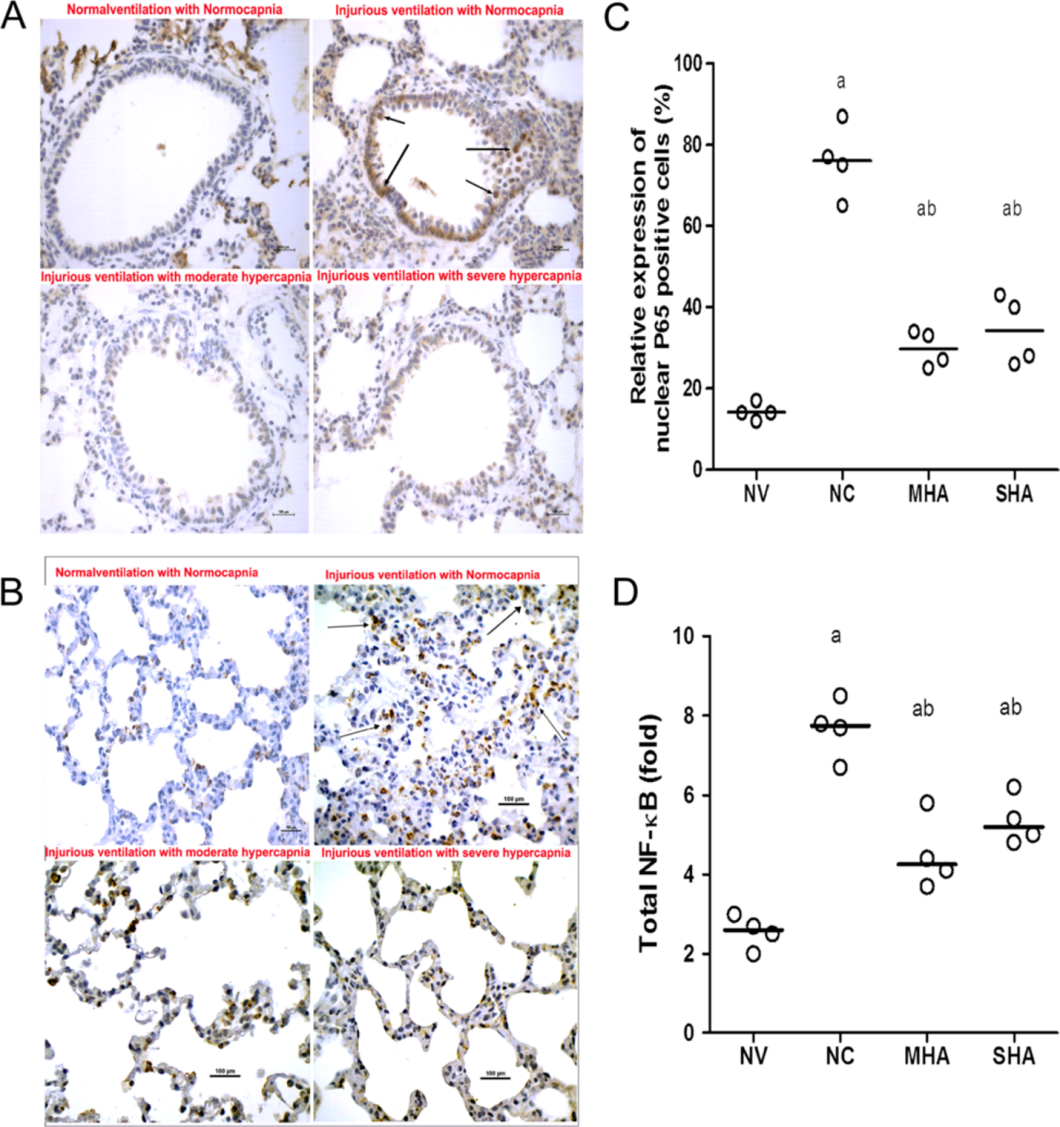
Lung Immunohistochemistry for NF-κB p65 protein expression in lung tissues. NV = normal-pressure ventilation with normocapnia; NC = high-pressure ventilation with normocapnia; MHA = moderate hypercapnic acidosis; SHA = severe hypercapnic acidosis. **(A and B)** arrows indicate the position of NF-κB p65 expression, which was only observed in the nuclei of airway epithelial cells and alveoli of HPV-treated animals. **(C)** Scatter plot of NF-κB p65 -positive (+) cells (%) in lung tissue. NF-κB expression also increased after 4 h of HPV compared to the normal ventilation group (p < 0.05), but this was attenuated by both moderate and severe hypercapnia. **(D)** ELISA analysis of total lung tissue NF-κB indicated that the NF-κB activity also increased after 4 h of HPV compared to the normal ventilation group but was attenuated by both moderate and severe hypercapnia. All panels represent a 400x magnification. Horizontal bars represent the median. *n = 4 for each group*. ^a^*p < 0.05 versus the NV group;*^*b*^*p < 0.001 versus the NC group.*

Most NF-κB signals were located in the alveoli and small airway epithelial cells and were mainly expressed in the nucleus (Figure 5A and B). The expression of NF-κB in lung tissues was significantly decreased in the HA groups compared with the normal ventilation group (Figure 5C, P < 0.05). Furthermore, HA significantly reduced total lung tissue NF-κB activity compared with the normal-ventilated group as evidenced by the ELISA assay (Figure 5D, P < 0.05). Western blot analysis for nuclear p65 also revealed an increase in expression in the NC group and a reduction in both HA groups (Figure 4A and B, P < 0.05). With 4 h of normocapnic HPV, IκB-α protein expression significantly decreased, but levels were relatively higher in both the moderate and severe hypercapnic groups (Figure 4A and D, P < 0.05).

## Discussion

Our study demonstrates that compared with high PaCO_2_ (130-150 mmHg) ventilation, rats receiving ventilation with a PaCO_2_ of 80-100 mmHg achieved better oxygenation with fewer histopathologic changes and less inflammatory injury. Furthermore, the hypercapnic acidosis induced by inhalational application of CO_2_ led to downregulation of NF-κB activity accompanied by a reduction in lung ICAM-1 expression.

This study was performed using a normal rat lung model, which does not reflect the same pathophysiology observed in humans or in acute respiratory distress syndrome (ARDS) [25]. As we know that the study by Sinclair [13] demonstrated that high tidal volume ventilation (25 cc/kg) for 4 hours produced a peak airway pressure about 33 cmH_2_O in eucapnic group, so we chose the high-pressure models used by others to induce VILI [24], which also caused impaired oxygenation and induced an acute lung inflammatory response. Moreover, we only observed the varying levels of PaCO_2_ and their impact on the development of lung injury induced by high-pressure ventilation, without clouding the results with other potential variables such as FiO_2_ and PEEP. In the current study, we chose a pressure control mode with 30 cmH_2_O PIP ventilation throughout the protocol, V_T_ decreased along with the development of lung injury and impaired oxygenation while PaCO_2_ increased correspondingly. Thus, this could reflect the same pathophysiology observed in the others research regarding VILI. Moreover, our study showed that acute hypercapnic acidosis was well-tolerated as long as perfusion and arterial oxygenation were maintained in the hypercapnic acidosis groups. This result likely occurred because inhaled CO_2_ improves gas exchange and ventilation-perfusion ratio matching [26] by reducing the heterogeneity of the pulmonary blood flow distribution and accordingly generates a higher PaO_2_ [27,28].

NF-κB is a key transcription factor in modulating various inflammatory genes including TNF-a, IL-1β and adhesion molecules. NF-κB activation also activates neutrophils accumulating in the lung tissue of ALI models [29-31]. However, even low-tidal volume ventilation activates inflammation (30). A study that looks at NF-κB translocation after VILI, and its down-regulation by NF-κB, it needs to demonstrate that this is a high stretch phenomenon, and does not occur with low stretch mechanical ventilation in the model also. Thus, we have a control group of animals who are either subject to normal pressure ventilation for four hours, or sham anaesthetized and non-ventilated animals. After 4 h of HPV, we found that NF-κB was mostly expressed in alveoli and small airway epithelial cells receiving only HPV. This observation was verified with an ELISA and western blot, both of which demonstrated that the NF-κB pathway in the lung tissue was markedly inhibited by hypercapnic acidosis. In the cytoplasm, the I-κB degradation prompts NF-κB to transfer into the nucleus, where it acts as a transcription factor regulating the expression of inflammatory mediators (TNF-α and IL-1β, etc.). These factors can be partially reversed by hypercapnic acidosis, which reveals that NF-κB should be an important factor in the process. Previous reports showed that hypercapnic acidosis reduced the severity of both mild and severe VILI by reducing NF-κB activation via a decrease in the breakdown of cytosolic I-κB inhibitory proteins; *in vitro* studies provided further support for this mechanism of action of hypercapnic acidosis [12]. However, our *in* vivo study found that HA abolished the decrease in densitometric lung tissue cytoplasmic IκBα concentrations induced by HPV. HA also inhibited HPV-induced up-regulation of the expression of ICAM-1 and MPO activity. Furthermore, Cummins also demonstrated reversible IKK-α nuclear localization in a CO_2_-dependent manner over a range of physiologic CO_2_ concentrations that was associated with an attenuation of LPS-induced NF-κB signaling and target-gene expression. This finding is consistent with CO_2_ affecting IKK-α and contributing to the attenuation of inflammation [32]. However, it is unclear whether CO_2_- or pH-mediated anti-inflammatory mechanisms contribute to lung protection in therapeutic hypercapnia. One study demonstrates that hypercapnia inhibits the release of interleukin-8 from stimulated leukocytes; such inhibition appears to be based on increased intracellular H^+^ and dependent on intracellular carbonic anhydrase [33]. These results suggest that the effects of hypercapnic acidosis on the NF-κB pathway may be complex and dependent on the milieu of unstable intermediates created by the interactions between hydrogen ions and CO_2_.

Although moderate hypercapnic acidosis is commonly observed when using protective ventilator strategies in experimental animal settings, our report is the first study on the effect of greater CO_2_ “doses” on VILI to our knowledge. Previous studies have shown that prophylactic maintenance of inhaled CO_2_ (12-25%) to maintain PaCO_2_ at a level of 80-100 mmHg reduced ischemia–reperfusion injury [5,22] and VILI [11,13]. However, whether a PaCO_2_ of 100 mmHg is the upper limit of the protective range of hypercapnia in VILI remains unknown. In the current study, although severe hypercapnic acidosis (PaCO_2_ at 130-150 mmHg) attenuated the severity of VILI, with prolonged ventilation until the end of the protocol, the oxygenation and histopathologic changes were progressively worse compared with moderate hypercapnic acidosis (PaCO_2_ at 80-100 mmHg). The protective effects of hypercapnia may be offset by a potential for adverse effects at higher levels. This possibility is supported by previous animal studies suggesting that protection from the adverse effects of brain ischemia was superior when the inhaled CO_2_ was kept at 6% rather than at 9% [34]. Of importance, severe hypercapnia induced by inhaling 15% CO_2_ has been proven to aggravate neurologic injury [35]. Furthermore, acute elevations in PaCO_2_ may induce intracellular acidosis and result in cardiovascular compromise, muscle weakness, increased intracranial pressure and central nervous system dysfunction [36]. It is obvious that acidosis is a double-edged sword that may be difficult to apply in critically ill patients. Nevertheless, the results of our study show that induction of HA by the addition of carbon dioxide to the inspired gas may necessitate attention to the potential for adverse effects at higher levels of PaCO_2_ in patients with ARDS during mechanical ventilation. On the other hand, our studies must be viewed as hypothesis-generating and should be tested by intensive studies in preclinical models.

Despite its interesting results, this exploratory study was limited in several ways. First, the animals were anesthetized with an intraperitoneal injection of pentobarbital sodium alone, which may not have reached the depth of anesthesia corresponding to clinical practice. Further studies should avoid this inappropriate anesthesia. Second, the pulmonary vasoconstriction and lung mechanics (i.e., plateau pressures, compliance, etc.) were not measured, which limited the interpretation of the specific effects of hypercapnic acidosis on pulmonary and systemic hemodynamic parameters. Third, the duration of the ALI models was limited to 4 h of mechanical ventilation and was too short to extrapolate to clinical practice. The results indicate that it is reasonable to believe that the acidosis generated by acute hypercapnia may be an important factor in acute models of VILI. Further study should be performed to evaluate the effects of hypercapnia in ALI models of considerably longer duration.

## Conclusion

This study demonstrates that an increased level of carbon dioxide has a protective effect against VILI in rats. Animals exposed to moderate hypercapnia (a PaCO_2_ of 80-100 mmHg) remained in a more favorable condition and had less histopathologic changes and inflammatory injury than animals with severe hypercapnia (a PaCO_2_ of 130-150 mmHg). The protective mechanism is likely associated with the inhibition of NF-κB expression during high pressure stretch.
